# Kinetic Model for Signal Binding to the Quorum Sensing Regulator LasR

**DOI:** 10.3390/ijms140713360

**Published:** 2013-06-27

**Authors:** Anetta Claussen, Tim Holm Jakobsen, Thomas Bjarnsholt, Michael Givskov, Martin Welch, Jesper Ferkinghoff-Borg, Thomas Sams

**Affiliations:** 1 Biomedical Engineering, Department of Electrical Engineering, Ørsteds Plads 349, Technical University of Denmark, Kongens Lyngby DK-2800, Denmark; E-Mail: anetta.claussen@gmail.com; 2 Costerton Biofilm Center, Department of International Health, Immunology, and Microbiology, Panum Institute, University of Copenhagen, Blegdamsvej 3B, Copenhagen DK-2200, Denmark; E-Mails: tholm@sund.ku.dk (T.H.J.); tbjarnsholt@sund.ku.dk (T.B.); mgivskov@sund.ku.dk (M.G.); 3 Singapore Centre on Environmental Life Sciences Engineering, Nanyang Technological University, Singapore 637551, Singapore; 4 Department of Biochemistry, University of Cambridge, Hopkins Building, Downing Site, Cambridge CB2 1QW, UK; E-Mail: mw240@cam.ac.uk; 5 Center for Biological Sequence Analysis, Department of Systems Biology, Building 301, Technical University of Denmark, Kongens Lyngby DK-2800, Denmark; E-Mail: jesperfb@cbs.dtu.dk

**Keywords:** quorum sensing, LasR, *Pseudomonas aeruginosa*, OdDHL, C12-HSL, signal molecule, ligand

## Abstract

We propose a kinetic model for the activation of the *las* regulon in the opportunistic pathogen *Pseudomonas aeruginosa*. The model is based on *in vitro* data and accounts for the LasR dimerization and consecutive activation by binding of two OdDHL signal molecules. Experimentally, the production of the active LasR quorum-sensing regulator was studied in an *Escherichia coli* background as a function of signal molecule concentration. The functional activity of the regulator was monitored via a GFP reporter fusion to *lasB* expressed from the native *lasB* promoter. The new data shows that the active form of the LasR dimer binds two signal molecules cooperatively and that the timescale for reaching saturation is independent of the signal molecule concentration. This favors a picture where the dimerized regulator is protected against proteases and remains protected as it is activated through binding of two successive signal molecules. In absence of signal molecules, the dimerized regulator can dissociate and degrade through proteolytic turnover of the monomer. This resolves the apparent contradiction between our data and recent reports that the fully protected dimer is able to “degrade” when the induction of LasR ceases.

## 1. Introduction

Quorum sensing (QS) relies on a cell-cell signaling system by which bacteria keep track of the density of the population. The quorum sensors, which also function as regulators of gene expression, enable the bacteria to control gene expression in relation to the population cell density and, accordingly, undergo collective phenotypic changes [1,2]. In Gram-negative bacteria, the quorum sensing regulatory system consists of a signal molecule synthase and a transcriptional regulator, referred to as LuxI and LuxR homologues, respectively. The signal molecules are acyl homoserine lactones (AHL). At low population density, signal molecules are present at concomitantly low levels. However, as the population density increases, the signal molecules accumulate and bind to the cognate transcriptional regulator inducing further transcription of the *luxI* homologue. This results in a rapid amplification of the signal [3–6].

Many LuxR homologues, including LasR of *Pseudomonas aeruginosa*, share the property that dimerization of the protein, necessary for transcriptional activation of target promoters, leads to significant protection against proteolytic degradation [7–15]. In a recent study, Sappington *et al.* showed that, in the cellular environment, the activated LasR-OdDHL complex is fully protected and that LasR and OdDHL (3-oxo-C_12_-HSL) readily dissociate and that LasR can remain in a conformation, which is capable of reassociating with signal molecules [16]. We shall take this observation as the starting point for our kinetic modeling by assuming that the active forms of the regulator are in quasistatic equilibrium and by assuming that the LasR fits the normal picture for transcription factors, *i.e*., relatively stable dimers *versus* degradable monomers.

Experimentally, we study the properties of LasR binding to predetermined concentrations of the signal molecule, OdDHL, in the *Escherichia coli* strain, MH155, hosting pMHLAS [17]. This plasmid encodes *lasR* under the control of *P_lac_* and the LasR-responsive, *P_lasB_*, driving expression of *lasB-gfp*(ASV). This leads to production of the observable unstable variant of green fluorescent protein (GFP(ASV)) [18]. The strain constitutes an experimental playground for examining the dimerization of LasR and the binding of the regulator to predetermined levels of OdDHL.

## 2. Results

**Figure 1:**
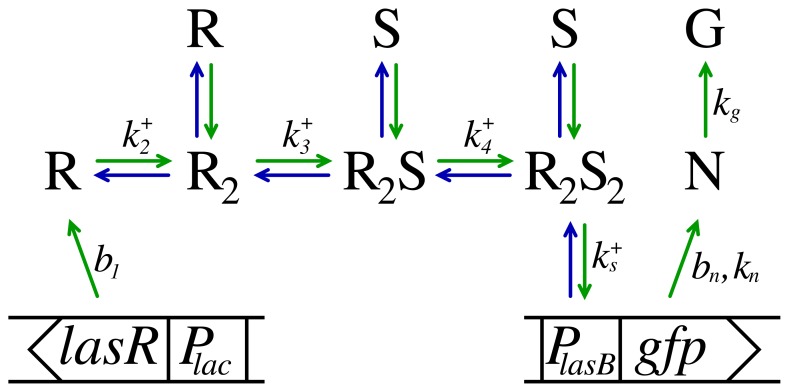
Schematic diagram of the functional components of the monitor strain used to measure the LasR - OdDHL kinetic response. In the diagram, the LasR regulator is denoted as R and the signal molecule, OdDHL, is denoted as S. On-rate constants for each process are indicated in the figure. The *lac*-*lasR* construct ensures constitutive production of LasR at rate *b*_1_. The regulator decays rapidly at rate λ_1_ or binds another R, forming a slowly decaying dimer, R_2_. When signal molecules are present, two signal molecules bind cooperatively to the dimer, which retains the slow proteolytic decay rate. The *lasB*-*gfp*(ASV) reporter fusion is used to monitor the R_2_S_2_ concentration and leads to induction of the reporter GFP(ASV), which matures into its measurable fluorescent form at rate *k_g_*.

**Figure 2:**
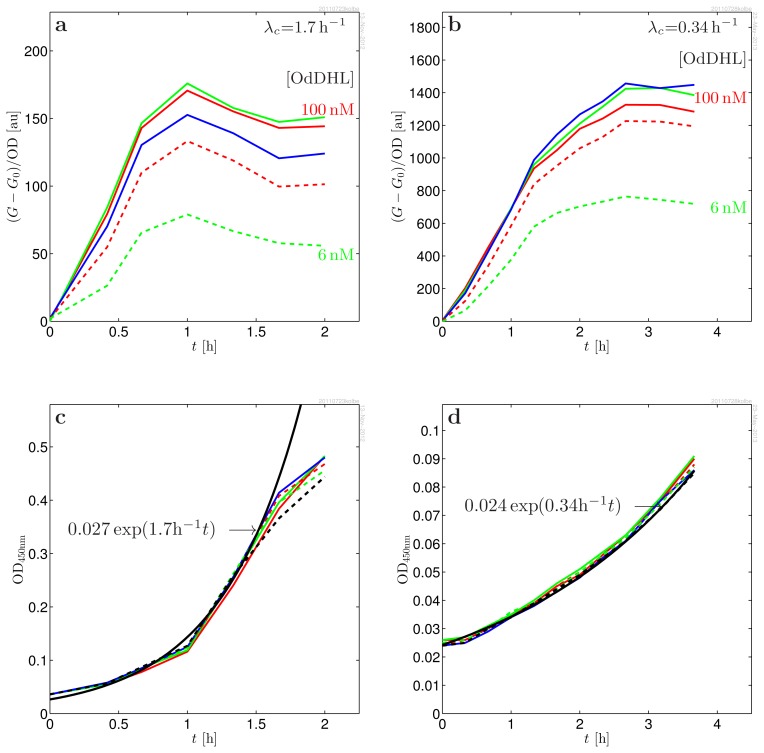
(**a**, **b**): Induced response of MH155 [*Plac-lasR PlasB-gfp*(ASV)] to predetermined concentrations of signal molecules *s* = [OdDHL] = 100 nM (red), 50 nM (green), 25 nM (blue), 12 nM (dash red), 6 nM (dash green). The response is baseline subtracted and normalized to the OD_450 nm_; (**c**, **d**): OD_450nm_ used for normalization of the fluorescence data. The deduced exponential growth rates are used when modeling the data. The fast growth (**a**, **c**) was obtained using glucose as carbon source and Casamino acids as amino acid source. The slow growth (**b**, **d**) was obtained with glycerol as carbon source and l-Leucine as sole amino acid source.

**Figure 3:**
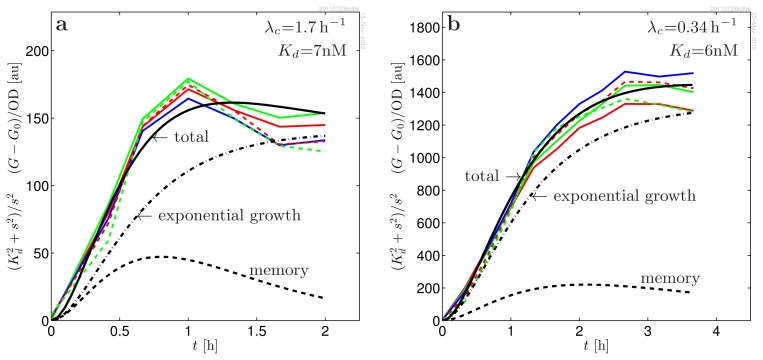
Data collapse of the induced response of MH155 [*Plac-lasR PlasB-gfp*(ASV)] to predetermined concentrations of signal molecules, *s* = [OdDHL] = 100 nM (red), 50 nM (green), 25 nM (blue), 12 nM (dash red) and 6 nM (dash green), at two different growth rates. The data collapse is obtained by dividing out the signal molecule switch, $s^{2}/(K_{d}^{2}+s^{2})$, as indicated in the ordinate label. Practically, the same *K_d_* is observed in the least square fitting at the two very different growth rates. The time structure is completely determined by the production and maturation of the unstable variant of green fluorescent protein, GFP(ASV), and is independent of the signal molecule concentration. This favors a picture where the regulator dimerization occurs before its binding to the signal molecules, the kinetics is fully cooperative, and the LasR dimer is fully protected already before ligand binding. The model curves are produced with the parameters in Table 1. In the model curves (black), the total yield (full) has been separated into the memory of past growth conditions (dash) and the component from exponential growth (dash-dotted), described in Equation (35).

When preparing a series of *in vitro* experiments to investigate the binding of LasR to OdDHL (reported as activation of *lasB-gfp* expression as illustrated in Figure 1), we observed that cultures brought to exponential growth directly from overnight inocula grown in the absence of OdDHL had a much larger response to the introduction of OdDHL than inocula that had been growing exponentially for a long time. Only after many generations in exponential growth does this “memory” fade. This led us to consider whether the active form of LasR may be stable, which could lead to elevated regulator concentrations at the lower growth rates attained in overnight cultures. However, it was not clear whether the memory of the initial condition reflected an elevated dimer level or was due to an accumulation of plasmids. In fact, plasmid concentration, as well as transcription rates can be strongly dependent on growth rates [19–22]. On top of this, there are significant uncertainties in interpretation of the GFP(ASV) reporter scaling when growth rate changes. We shall therefore not attempt to make conclusions concerning a possible accumulation of dimers *versus* accumulation of plasmids in the close-to-stationary phase based on the absolute scale of the responses in different growth conditions. Instead, we modeled and analyzed the detailed shape and scaling of the response to introduction of OdDHL at two very different growth rates (λ*_c_* = 0.34 h^−1^ and 1.7 h^−1^). In these experiments, the cultures were maintained at exponential growth for many generations prior to introduction of the signal molecules. Below, we shall briefly summarize the properties of the model, while the details are given in the methods section. The model is based on observations from Figures 2 and 3 and on a the recent findings by Sappington *et al.* [16]. (a) Inside the living cell, the active form of LasR readily associates and dissociates from the ligand [16]. This means that we can assume quasistatic equilibrium between the active conformation of LasR and the LasR-OdDHL complex; (b) The activated (ligand bound) regulator is fully protected against proteases [16]; (c) From the data collapses in Figure 3, we see that the response to introduction of signal molecules follows second order cooperative kinetics, $s^{2}/(K_{d}^{2}+s^{2})$, *s* = [OdDHL]. This suggests that the active form is a dimer and that it binds two signal molecules cooperatively, *i.e*., dimerization drives ligand binding. (The *s*^2^*/*(*K_d_* + *s*)^2^ kinetics when ligand binding is non-cooperative or when ligand binding drives dimerization is not compatible with our data;) (d) From the same figure, we see that the shape of the response is independent of the ligand concentration, *i.e*., the response time is independent of ligand concentration. This leads to the conclusion that the active conformation of the regulator has the same half-life as the ligand bound regulator. (In fact, we shall see that the shape of the response is fully accounted for by the properties of the GFP(ASV) reporter alone. This means that, within the resolution of the experiment, the response of *lasB* to introduction of signal molecules is instantaneous;) (e) When LasR production is switched off, the active conformation survives in the cell for 20 min in absence of OdDHL [16]. This fairly rapid disappearance in combination with the stability of the active conformation suggests that the disappearance goes through the monomer channel arising from dissociation of the dimer. In the Methods, we shall see that this leads us to conclude that the monomer is transient. When the production is switched off, the condition for transient monomer breaks down, and the degradation through the monomer channel opens up.

We shall make a rough summary of the model. After many generations at constant growth rate, λ*_c_*, steady state is reached for the total dimerized regulator level, *r_d_*,

(1) $$r_{d}=[{\text{R}}_{2}]+[{\text{R}}_{2}\text{S}]+[{\text{R}}_{2}{\text{S}}_{2}]=\frac{b_{1}R_{t}}{2\lambda_{c}}$$

This is a product of half the monomer production rate, *b*_1_, and the plasmid concentration, *R_t_*, diluted by the growth rate, λ*_c_*.

When no signal molecules are present, all dimers are in the free form, R_2_. As signal molecules are added at concentration, *s*, the concentration of dimerized activated regulator quickly adjusts to:

(2) $$r_{4}=r_{d}\frac{s^{2}}{K_{d}^{2}+s^{2}}=\frac{b_{1}R_{t}}{2\lambda_{c}}\frac{s^{2}}{K_{d}^{2}+s^{2}}$$

This is a product of the maximal dimer level and the switch in the signal molecule concentration, *s*, at the effective dissociation constant, *K_d_*.

Following a change in the signal molecule concentration, the activated dimer concentration adjusts rapidly. When the growth rate, transcription rate or plasmid density changes the dimer concentration exponentially, changes to its new value at rate, λ*_c_* (as described in Equation (22)). The activated dimer level may be written as a sum of a static growth term and a “memory” term:

(3) $$r_{4}=[{\text{R}}_{2}{\text{S}}_{2}]=\frac{b_{1}R_{t}}{2\lambda_{c}}\;(1+m_{d}\;{\text{e}}^{-\lambda_{c}t})\;\frac{s^{2}}{K_{d}^{2}+s^{2}}$$

where *m_d_* is the initial condition resulting from past growth conditions. This memory term describes the elevated response for overnight cultures, as well as the “overshoot” observed in cultures that have not been kept in exponential growth mode sufficiently long to bring the plasmid and dimer concentrations to equilibrium. If the dimer/plasmid level achieved in stationary phase is much larger than the new asymptotic dimer level, it may take many generations to “forget” the stationary phase level. The *s*-dependence of *r*_4_ is accounted for by the fully cooperative Hill-factor, and the *t*-dependence is described by the exponential memory term in Equation (3).

A detailed comparison with the experimental data, which requires a description of the properties of the GFP probe, as well, is shown in Figure 3. Here, we see that the measured responses for exponential inocula are well accounted for by the model.

## 3. Discussion

The new data shows that the response to introduction of signal molecules follows second order cooperative kinetics. In addition, we observe an identical fast response time to different levels of OdDHL. Were the two dimer forms not both long-lived, we would have observed a fast response for low levels of signal molecule and a slower response for high levels of signal molecule.

The evidence that the active form of LasR is a dimer is in line with size-exclusion chromatography and dynamic light scattering data from activated LasR in solution [15]. Further, the reported crystal structure of the ligand binding domain of LasR is a dimer binding two ligand molecules [24].

However, Sappington *et al.* also observe that when the production of regulator is stopped, in the absence of signal molecules, the active form of the regulator disappears is around 20 min. At first sight, this appears in contradiction with our finding, *i.e*., that the dimer form of the regulator is protected against proteases. We demonstrate that this apparent contradiction can be resolved in our kinetic model: when production of regulator is turned off, the condition for transient monomer is no longer satisfied. The dimerized regulators are then free to disappear through disassociation into monomers, followed by degradation at the faster monomer degradation rate.

We therefore propose a very conventional picture of the LasR regulator: the regulator monomer has a rapid proteolytic turnover; the dimerized regulator is protected against proteases and remains protected against proteases as it is activated through cooperative binding of two signal molecules. Below the threshold for transient monomer, the (LasR)_2_ dimer degrades rapidly through the monomer channel.

We shall see in the Methods that getting access to see these properties of the LasR regulator function relies on being in the limit of transient monomer, where the timescale from monomer degradation does not occlude the picture. Therefore, one cannot expect to observe the underlying cooperative kinetics and the response time invariance as clearly with the weaker native *P_lasR_* promoter in a single copy in the chromosome of *P. aeruginosa*.

## 4. Methods

### 4.1. Kinetic Model

The kinetic equations for the model in Figure 1 are now established. The constitutive transcription of regulator molecules, R, with concentration, *r*_1_, is given by Equation (4) and is proportional to the concentration, *R_t_*, of *lasR* sites and the lasR transcription rate, *b*_1_. This results in a steady production of LasR regulator, which rapidly decays at rate, λ_1_, or forms dimers. Cell division is included through addition of the growth rate, λ*_c_*, to the proteolytic decay.

The observed second order cooperative response indicates that LasR dimerizes (5) before binding OdDHL. In the activation of the regulator through binding of two signal molecules, Equations (6) and (7), is in quasistatic equilibrium.

In all, the regulator formation and binding to the ligand is described by the kinetic equations:

(4) $$\frac{dr_{1}}{dt}=b_{1}R_{t}+2k_{2}^{-}r_{2}-2k_{2}^{+}r_{1}^{2}-(\lambda_{1}+\lambda_{c})r_{1}$$

(5) $$\frac{dr_{2}}{dt}=k_{2}^{+}r_{1}^{2}+k_{3}^{-}r_{3}-2k_{3}^{+}r_{2}s-(k_{2}^{-}+\lambda_{2}+\lambda_{c})r_{2}$$

(6) $$\frac{dr_{3}}{dt}=2k_{3}^{+}r_{2}s+2k_{4}^{-}r_{4}-k_{4}^{+}r_{3}s-(k_{3}^{-}+\lambda_{3}+\lambda_{c})r_{3}$$

(7) $$\frac{dr_{4}}{dt}=k_{4}^{+}r_{3}s-(2k_{4}^{-}+\lambda_{4}+\lambda_{c})r_{4}$$

The sensor for the activated dimer level is the *lasB*-*gfp*(ASV) translational fusion. The rate of binding R_2_S_2_ to the *lasB* promoter site is proportional to the concentration of free sites, *S_f_* = *S_t_* − *S_a_*, and the concentration of activated dimers, while the dissociation rate is proportional to the number of occupied sites, *S_a_* (8). The production (9) of non-mature GFP(ASV) is the sum of a small background production proportional to the number of free sites and an induced production proportional to the number of occupied promoter sites. The maturation of GFP into its fluorescent form is described by Equation (10).

(8) $$\frac{dS_{a}}{dt}=k_{S}^{+}r_{4}(S_{t}-S_{a})-(k_{S}^{-}+\lambda_{c})S_{a}$$

(9) $$\frac{dn}{dt}=b_{n}S_{t}+(k_{n}-b_{n})S_{a}-(k_{g}+\lambda_{n}+\lambda_{c})n$$

(10) $$\frac{dg}{dt}=k_{g}n-(\lambda_{g}+\lambda_{c})g$$

In order to emphasize its role, the growth rate, λ*_c_*, is explicitly included throughout.

Leveau *et al.* find that the proteolytic decay of GFP is Michaelis-Menten limited [23] for some of the very short lived variants introduced by Andersen *et al.* [18], but not important in GFP(ASV). We have therefore used the linear description in Equations (9) and (10) in our model.

#### 4.1.1. Driven Systems

The signal sensor of the driven system in Figure 1 consists of an input channel and four regulatory units with concentrations, *r*_1_ = [R], *r*_2_ = [R_2_], *r*_3_ = [R_2_S] and *r*_4_ = [R_2_S_2_]. Now, what comes in must go out. The entry channel is the production of monomer regulator at rate *b*_1_*R_t_*, *i.e*., the product of the production rate per plasmid and the plasmid concentration. The exit channels are the dilution, λ*_c_*, from cell growth and the proteolytic degradation, λ*_i,_i* = 1*, ...,* 4, of each form of the regulator. The total regulator balance is then:

(11) $$\frac{dr}{dt}=b_{1}R_{t}-\lambda r$$

(12) $$r=r_{1}+2r_{2}+2r_{3}+2r_{4}$$

(13) $$\lambda =\frac{r_{1}}{r}(\lambda_{1}+\lambda_{c})+\frac{2r_{2}}{r}(\lambda_{2}+\lambda_{c})+\frac{2r_{3}}{r}(\lambda_{3}+\lambda_{c})+\frac{2r_{4}}{r}(\lambda_{4}+\lambda_{c})$$

Since the activated regulator, R_2_S_2_, is well protected against proteases, we have λ_4_ = 0. Further, the equilibration between the dimer forms is rapid [16] and controlled by the signal molecule concentration. Finally, in Figure 3a,b, we observe that response time to changes in signal molecule concentration is independent of its concentration. If the non-activated forms of the regulator, R_2_ and R_2_S, had faster degradation than the active R_2_S_2_, this would lead to a faster timescale at lower concentrations of S. We therefore conclude that the proteolytic degradation rate of all dimer forms are similar, *i.e*., λ_2_ = λ_3_ = λ_4_ = 0.

In absence of signal molecules, the active conformation of the regulator was observed to disappear in around 20 min after the production was turned off [16]. We are therefore in need for a fast degradation channel. This means that the monomer degradation is rapid and that the active conformation degrades primarily through disassociation, followed by monomer degradation. The dissociation, as well as the degradation rate need to be sufficient to account for the fast disappearance of the dimer. The dissociation constant for the ligand-free dimer is therefore large. Now, an apparent conflict appears: The higher degradation rate for the monomer should lead to shorter response time for lower signal molecule concentrations. Since this is not observed in our experiment, the influence of λ_1_ in the weighted average (13) must be small. This means that the monomer is transient, *r*_1_λ_1_ ≪ 2(*r*_2_ + *r*_3_ + *r*_4_)λ*_c_*, in our experiment and that this condition is not satisfied in the experiment of Sappington *et al.*, where the monomer production is switched off. The detailed kinetics elaborated below reveals an accelerated degradation of the regulator through the monomer channel as the concentration decreases. This is in excellent agreement with the reported experiment [16].

#### 4.1.2. Ligand Binding

During build-up, the balance between dimer forms can be assumed in quasistatic equilibrium. Equations (6) and (7) then lead to:

(14) $$r_{4}=\frac{s^{2}}{s^{2}+2K_{4}s+K_{3}K_{4}}r_{d}$$

In the limit of cooperative ligand binding (*K*_4_ ≪ *K*_3_):

(15) $$r_{4}=\frac{s^{2}}{s^{2}+K_{d}^{2}}r_{d}$$

(16) $$K_{d}^{2}=K_{3}K_{4}$$

*i.e*., cooperative binding with Hill coefficient 2. As can be seen from the data collapses in Figure 3, this gives a good description of the *s*-dependence of the data.

#### 4.1.3. Turnover of Dimer Variants

In order to determine whether R_2_ with no ligand bound could be short-lived, we will again consider what would happen if the turnover of the dimer, R_2_, were significantly faster than the turnover of the activated dimer, R_2_S_2_. When the ligand binding is cooperative:

(17) $$\lambda_{d}=\frac{r_{2}}{r_{d}}\lambda_{2}+\frac{r_{3}}{r_{d}}\lambda_{3}+\frac{r_{4}}{r_{d}}\lambda_{4}$$

(18) $$=\frac{K_{d}^{2}}{K_{d}^{2}+s^{2}}\lambda_{2}+\frac{s^{2}}{K_{d}^{2}+s^{2}}\lambda_{4}$$

We already know that R_2_S_2_ is well protected against proteases [16]. If λ_2_ were much larger than the growth rate, Equation (18) would cause the response time to be fast when only small amounts of signal molecules were added and slower when large amounts were added. Since the data collapses in Figure 3 show that the response time is independent of the signal molecule concentration, we conclude that the two dimer variants are both well protected against proteases, *i.e*., λ_2_ = λ_4_ = 0.

#### 4.1.4. Regulator Formation

Assuming that on/off rates are rapid relative to degradation rates, the monomer formation (4) is to a good approximation in quasistatic equilibrium. This leads to:

(19) $$r_{1}=\frac{\lambda_{1}}{4k_{2}^{+}}\;\left(\sqrt{1+\frac{8k_{2}^{+}(b_{1}R_{t}+2k_{2}^{-}r_{2})}{\lambda_{1}^{2}}}-1\right)$$

where we have used λ*_c_* ≪ λ_1_.

We get the dimer build-up by adding up Equations (5)–(7), describing the formation of the dimer variants:

(20) $$\frac{dr_{d}}{dt}=k_{2}^{+}r_{1}^{2}-k_{2}^{-}r_{2}-\lambda_{c}r_{d}$$

(21) $$r_{d}=r_{2}+r_{3}+r_{4}$$

where we used that, all dimer forms are well protected against proteases.

In the limit of transient monomer, $8k_{2}^{+}b_{1}R_{t}\gg \lambda_{1}^{2}$, Equations (19) and (20) result in:

(22) $$\frac{dr_{d}}{dt}=\frac{b_{1}R_{t}}{2}-\lambda_{c}r_{d}$$

describing the population of the dimer states. The monomer degradation does not appear in Equation (22), since there is limited access to this channel when the monomer is transient. When the growth conditions change, the static dimer concentration:

(23) $$r_{d}=\frac{b_{1}R_{t}}{2\lambda_{c}}$$

is approached exponentially at rate λ*_c_*. Notably, the total dimer concentration, *r_d_*, is independent of the signal molecule concentration. When signal molecules are added, the new balance between the active form, R_2_, and the activated form, R_2_S_2_, is quickly established. This results in Equation (3).

#### 4.1.5. The Monitor

In order to connect to the experiment, the monitor needs to be described. The binding of the activated dimer to the *lasB* promoter site, Equation (8), is solved in the quasistatic limit. This results in:

(24) $$S_{a}=\frac{r_{4}}{r_{4}+K_{S}}S_{t}\overset{r_{4}\ll K_{S}}{\approx }\frac{r_{4}}{K_{S}}S_{t}$$

(25) $$K_{S}=\frac{k_{S}^{-}+\lambda_{c}}{k_{S}^{+}}$$

Since very different maximal responses for exponential inocula at different growth rates (Figure 3) and overnight cultures lead to a similar observed dissociation constant, we may assume that *r*_4_ ≪ *K_S_*. This results in linear scaling with the activated dimer concentration.

The induced GFP production described by Equations (9) and (10) is linear in the activated operator concentration, *S_a_*:

(26) $$g(t)-g_{0}=h_{g}(t)*h_{n}(t)*S_{a}(t)$$

(27) $$h_{n}(t)=(k_{n}-b_{n})\;\text{exp}(-\Lambda_{n}t)$$

(28) $$h_{g}(t)=k_{g}\;\text{exp}(-\Lambda_{g}t)$$

(29) $$\Lambda_{n}=k_{g}+\lambda_{n}+\lambda_{c}$$

(30) $$\Lambda_{g}=\lambda_{g}+\lambda_{c}$$

where ‘*’ denotes convolution with the impulse responses, *h_n_* and *h_g_*. This introduces a delay and further suppression of the response at high growth rates.

#### 4.1.6. The Measured Response

In experiments where the culture is kept at a fixed growth rate for many generations before adding signal molecules, the measured, OD-normalized, response is proportional to:

(31) $$g(t)-g_{0}=\frac{S_{t}}{K_{S}}\frac{k_{n}-b_{n}}{\Lambda_{n}}\frac{k_{g}}{\Lambda_{g}}\frac{b_{1}R_{t}}{2\lambda_{c}}\frac{s^{2}}{K_{d}^{2}+s^{2}}f(t)$$

The time structure lies in the step response for the GFP monitor, defined as:

(32) $$f(t)=1-\frac{\Lambda_{n}}{\Lambda_{n}-\Lambda_{g}}\;\text{exp}(-\Lambda_{g}t)+\frac{\Lambda_{g}}{\Lambda_{n}-\Lambda_{g}}\;\text{exp}(-\Lambda_{n}t)$$

conveniently normalized to unity at large *t*. The memory term has the same form, except, with a different form factor:

(33) $$f_{m}(t)=\frac{\Lambda_{n}\Lambda_{g}}{\left(\Lambda_{n}-\lambda_{c})\;(\Lambda_{n}-\Lambda_{g})\;(\Lambda_{g}-\lambda_{c}\right)}((\Lambda_{n}-\Lambda_{g}){\text{e}}^{-\lambda_{c}t}+(\Lambda_{g}-\lambda_{c}){\text{e}}^{-\Lambda_{n}t}+(\Lambda_{c}-\lambda_{n}){\text{e}}^{-\Lambda_{g}t})$$

which includes the decay of the memory (initial condition) in (3). This results in an OD-normalized induced response:

(34) $$\frac{G(t)-G_{0}(t)}{\text{OD}}=\;\;\;\frac{A}{\Lambda_{n}\Lambda_{g}\lambda_{c}}\;\;\;\frac{s^{2}}{K_{d}^{2}+s^{2}}\;(f(t)+m_{d}\;f_{m}(t))$$

to be compared to data. The constant, *A*, contains the (arbitrary) fluorescence counter normalization and the remaining (growth rate dependent) constants. By multiplying this equation by $(K_{d}^{2}+s^{2})/s^{2}$, we get:

(35) $$\frac{G(t)-G_{0}(t)}{\text{OD}}\;\;\;\frac{K_{d}^{2}+s^{2}}{s^{2}}=\;\;\;\frac{A}{\Lambda_{n}\Lambda_{g}\lambda_{c}}\;(f(t)+m_{d}\;f_{m}(t))$$

where we observe that the rhs is independent of the ligand concentration. The *K_d_* dependence can therefore be fitted separately and leads to the data collapse in Figure 3. This leaves λ*_n_* + *k_g_*, λ*_g_*, *A* and *m_d_* to be fitted to the collapsed data sets in Figure 3a,b. The resulting parameters are given in Table 1.

#### 4.1.7. Decay via the Monomer Channel

Sappington *et al.* recently pointed out that in the cellular environment, the active form of the regulator readily binds to and dissociates from the signal molecule [16]. Further, in a monitor strain where induction of LasR can be switched off, they demonstrate that LasR is able to survive in its active form without the presence of OdDHL, though not for more than 20 min.

Our findings show that the active form is a dimer, R_2_, which cooperatively binds two ligand molecules, forming R_2_S_2_, and require both forms to have a vanishing proteolytic degradation rate. How can we understand the apparent contradiction that, at the same time, R_2_ is not subject to proteolytic degradation and yet disappears in 20 min?

Within the conventional model, the answer is surprisingly straight forward: When there are no signal molecules present, the R_2_ dimers are free to dissociate into monomers, which are targeted for rapid proteolytic degradation. Due to the second order nature of the dimer formation, this process speeds up as the production of the regulator is switched off. Thus, in the absence of signal molecules, the dimers appear to be degrading, even though they are disassociating, and only subsequently degraded at the monomer degradation rate. At high signal molecule concentration, the dimers are all in the R_2_S_2_ form and do not have access to the monomer channel.

Let us write this down formally. When the production of the regulator is switched off, we have:

(36) $$\frac{dr_{1}}{dt}=2k_{2}^{-}r_{2}-2k_{2}^{+}r_{1}^{2}-(\lambda_{1}+\lambda_{c})r_{1}$$

(37) $$\frac{dr_{2}}{dt}=k_{2}^{+}r_{1}^{2}-(k_{2}^{-}+\lambda_{2}+\lambda_{c})r_{2}$$

in the absence of signal molecules. In the quasistatic limit of either of these equations, we get:

(38) $$\begin{array}{l} \frac{dr_{1}}{dt}=-\frac{\frac{1}{4}\frac{2(\lambda_{1}+\lambda_{c})}{\lambda_{2}+\lambda_{c}}K_{2}+r_{1}}{\frac{1}{4}K_{2}+r_{1}}\;\;\;\frac{\lambda_{2}+\lambda_{c}}{2}\;\;\;r_{1}\\ \approx \{\begin{array}{ll} -\frac{\lambda_{2}+\lambda_{c}}{2}r_{1} & ,\;r_{1}>\frac{1}{4}\frac{2(\lambda_{1}+\lambda_{c})}{\lambda_{2}+\lambda_{c}}K_{2}\\ -(\lambda_{1}+\lambda_{c})\;r_{1} & ,\;r_{1}<\frac{1}{4}K_{2} \end{array} \end{array}$$

and similarly:

(39) $$\begin{array}{l} \frac{dr_{2}}{dt}=-\frac{\frac{1}{4}\frac{2(\lambda_{1}+\lambda_{c})}{\lambda_{2}+\lambda_{c}}\sqrt{K_{2}}+\sqrt{r_{2}}}{\frac{1}{4}\sqrt{K_{2}}+\sqrt{r_{2}}}\;\;\;\frac{\lambda_{2}+\lambda_{c}}{2}\;\;\;r_{2}\\ \approx \{\begin{array}{ll} -(\lambda_{2}+\lambda_{c})\;r_{2} & ,\;r_{2}>\frac{1}{16}{\left(\frac{2(\lambda_{1}+\lambda_{c})}{\lambda_{2}+\lambda_{c}}\right)}^{2}K_{2}\\ -2(\lambda_{1}+\lambda_{c})\;r_{2} & ,\;r_{2}<\frac{1}{16}K_{2} \end{array} \end{array}$$

**Figure 4:**
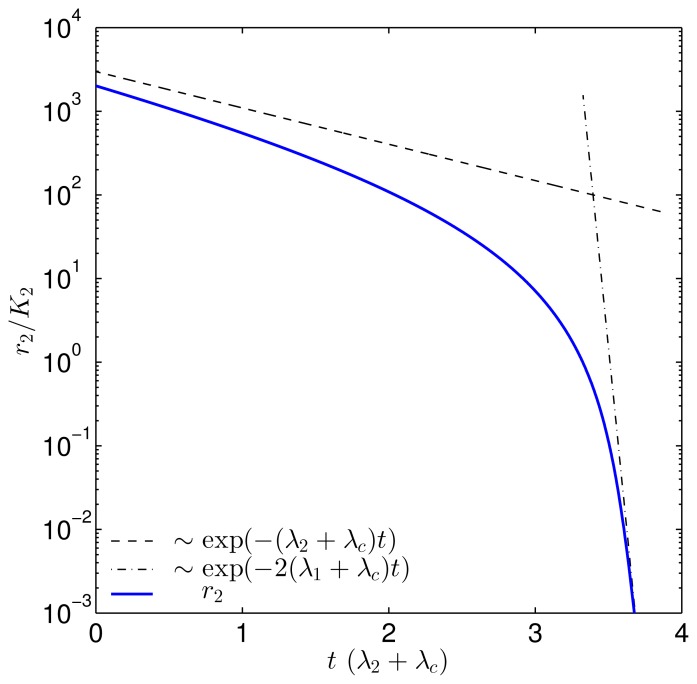
Plot showing the transition from direct degradation of the dimer to degradation through dissociation into rapidly degrading monomers, as shown in Equation (39). The (λ_1_ + λ*_c_*)*/*(λ_2_ + λ*_c_*) ratio, which determines the concentration at the transition, was set to 20. When the dimer concentration, *r*_2_, passes *K*_2_, the downward slope approaches the fast monomer degradation rate, which reflects the transition to degradation via the monomer channel.

This shows that the dimer, R_2_, dissociates and subsequently degrades through the open monomer channel at rate, 2(λ_1_ + λ*_c_*), when the concentration is low. In Figure 4, the solution to *r*_2_ is plotted with the asymptotes for (λ_1_ + λ*_c_*)*/*(λ_2_ + λ*_c_*) = 20. We clearly see the transition from slow degradation through the dimer channel to fast degradation through the monomer channel.

In the experiment by Sappington *et al.*, it is observed that the active conformation of non-ligand-bound LasR disappears at accelerated rate over time. This may well correspond to moving down the shoulder of the *r*_2_ curve in Figure 4.

### 4.2. Materials

Cultures of MH155 [pMHLAS] [17] were grown in fresh ABT minimal medium (B medium (Clark & Maaløe, 1967 [25]), containing 2.5 mg/L thiamine, 10% A10 (Clark & Maaløe, 1967 [25])), 0.5% glucose, and 0.5% casamino acids. This resulted in an exponential growth rate of λ*_c_* = 1.7 h^−1^. The low growth rate, λ*_c_* = 0.34 h^−1^, was obtained by substituting glucose by glycerol and the casamino acids by 0.5% l-leucine (Sigma Aldrich, St. Louis, MO, USA, CAS 61-90-5, L8000).

Following exponential growth at 37 °C with vigorous aeration (shaking at 200 rpm), the MH155 culture was diluted in fresh medium to OD_450nm_ = 0.01, measured on Shimadzu UV-1800 and distributed to preheated (37 °C) 100mL flasks containing 50mL two-fold dilution rows of OdDHL (Cayman Chemical, Ann Arbor, MI, USA, CAS 168982-69-2, item 10007895) starting at 100nM and leaving one flask without signal molecules for baseline determination.

Fluorescence from GFP(ASV) was measured using a Shimadzu RF-5301PC fluorimeter in quantitative raw data mode at λ_ex_ = 475 ±5 nm, λ_em_ = 515 ±5 nm, 4s integration time and amplification set to high. The induced response is the measured OD-weighted background subtracted response, (GFP − GFP_0_)/OD, where GFP and GFP_0_ are raw GFP counts with and without, respectively, signal molecules. (Detailed comparison to the model should only be made when the OD(t) and OD_0_(t) are identical.)

## 5. Conclusions

We established a conservative kinetic model for the regulator production and ligand binding in the *las* regulon of *Pseudomonas aeruginosa*. In the model, the regulator monomer has a rapid proteolytic turnover; the dimerized regulator is protected against proteases and remains protected against proteases as it is activated through cooperative binding of two signal molecules. We presented new data favoring this picture of the *las* regulon and resolved apparent contradictions between our data and other available data.

## Figures and Tables

**Table 1:** Parameters used in the model in the kinetic model.

Parameter	Value	
λ*_n_* + *k_g_*	(2.1 ± 0.3) h^−1^	degradation plus maturation (consistent with [18,23])
λ*_g_*, λ*_n_*	(0.7 ± 0.3) h^−1^	proteolytic decay for GFP (consistent with [18])
*K_d_*	(7 ± 2)nM	dissociation constant (agrees with [17])
*A*	2170 ± 200 [a.u.]	amplitude (Glucose, Casamino)
	1200 ± 200 [a.u.]	amplitude (Glycerol, l-leucine)
*m_d_*	0.9 ± 0.2	memory (Glucose, Casamino)
	0.3 ± 0.1	memory (Glycerol, l-leucine)
λ*_c_*	(1.69 ± 0.04) h^−1^	growth rate (Glucose, Casamino)
	(0.34 ± 0.03) h^−1^	growth rate (Glycerol, l-leucine)

*g*(*t*)		density normalized GFP response
*g*_0_		density normalized GFP response at *s* = 0
*G*(*t*)		measured fluorescence response
*G*_0_(*t*)		measured fluorescence response at *s* = 0
OD(*t*)		optical density at 450nm
*h_n_*(*t*), *h_g_*(*t*)		impulse response for GFP production and maturation
$k_{2}^{\pm }$		on/off rates for dimer formation
$k_{3}^{\pm },k_{4}^{\pm }$		on/off rates for ligand binding
*K*_2_	$k_{2}^{-}/k_{2}^{+}$	dimer dissociation constant
*K*_3_, *K*_4_	$k_{3}^{-}/k_{3}^{+},k_{4}^{-}/k_{4}^{+}$	ligand-dimer dissociation constants
*K_d_*	$\sqrt{K_{3}K_{4}}$	dissociation constant for cooperative ligand binding
*b*_1_	~ 1000 h^−1^	production rate of LasR per plasmid copy [21]
*b_n_*		background production of GFP(ASV)
*k_n_*	~ 1000 h^−1^	induced production rate of GFP per plasmid copy
*k_g_*	~ 1.5 h^−1^	maturation rate of GFP [23]
λ_1_	~ 20 h^−1^	R monomer degradation, [7]
λ_2_	~ 0 h^−1^	R_2_ degradation (this study)
λ_3_		R_2_S degradation, insensitive to this value
λ_4_	~ 0 h^−1^	degradation of R_2_S_2_[16]
λ*_d_*		averaged dimer degradation rate
Λ*_n_*	*k_g_* + λ*_n_* + λ*_c_*	GFP parameter
Λ*_g_*	λ*_g_* + λ*_c_*	GFP parameter
*R_t_*		*lac* promoter density (plasmid density)
*S_t_*		*lasB* promoter density (plasmid density)
*S_a_*		active *lasB* promoter density (plasmid density)
*S_f_*		free *lasB* promoter density (plasmid density)
*r*_1_, *r*_2_, *r*_3_, *r*_4_	[R], [R_2_], [R_2_S], [R_2_S_2_]	LasR monomer and dimer concentrations
*s*	[S]	Signal molecule concentration
*t*		time since addition of signal molecules
